# Human MicroRNA Targets

**DOI:** 10.1371/journal.pbio.0020363

**Published:** 2004-10-05

**Authors:** Bino John, Anton J Enright, Alexei Aravin, Thomas Tuschl, Chris Sander, Debora S Marks

**Affiliations:** **1**Computational Biology Center, Memorial Sloan-Kettering Cancer CenterNew York, New YorkUnited States of America; **2**Wellcome Trust Sanger InstituteCambridgeUnited Kingdom; **3**Laboratory of RNA Molecular Biology, The Rockefeller UniversityNew York, New YorkUnited States of America; **4**Department of Systems Biology, Harvard Medical SchoolBoston, MassachusettsUnited States of America

## Abstract

MicroRNAs (miRNAs) interact with target mRNAs at specific sites to induce cleavage of the message or inhibit translation. The specific function of most mammalian miRNAs is unknown. We have predicted target sites on the 3′ untranslated regions of human gene transcripts for all currently known 218 mammalian miRNAs to facilitate focused experiments. We report about 2,000 human genes with miRNA target sites conserved in mammals and about 250 human genes conserved as targets between mammals and fish. The prediction algorithm optimizes sequence complementarity using position-specific rules and relies on strict requirements of interspecies conservation. Experimental support for the validity of the method comes from known targets and from strong enrichment of predicted targets in mRNAs associated with the fragile X mental retardation protein in mammals. This is consistent with the hypothesis that miRNAs act as sequence-specific adaptors in the interaction of ribonuclear particles with translationally regulated messages. Overrepresented groups of targets include mRNAs coding for transcription factors, components of the miRNA machinery, and other proteins involved in translational regulation, as well as components of the ubiquitin machinery, representing novel feedback loops in gene regulation. Detailed information about target genes, target processes, and open-source software for target prediction (miRanda) is available at http://www.microrna.org. Our analysis suggests that miRNA genes, which are about 1% of all human genes, regulate protein production for 10% or more of all human genes.

## Abstract 2

This computational analysis provides evidence that as many as 10% of human genes are targets for regulation by small RNA molecules called microRNAs

## Introduction

### The Functions of MicroRNAs

In the past three years, several hundred novel genes encoding transcripts containing short double-stranded RNA hairpins, named microRNAs (miRNAs), were identified in plants and animals (Lee et al. 1993; Reinhart et al. 2000, 2002; Lagos-Quintana et al. 2001, 2002, 2003; Lau et al. 2001; Lee and Ambros 2001; Llave et al. 2002a; Mette et al. 2002; Mourelatos et al. 2002; Park et al. 2002; Ambros et al. 2003b; Aravin et al. 2003; Brennecke et al. 2003; Dostie et al. 2003; Grad et al. 2003; Houbaviy et al. 2003; Lai et al. 2003; Lim et al. 2003a, 2003b; Palatnik et al. 2003). More recently, miRNAs have also been identified in a large DNA virus, the Epstein Barr virus, and are likely to be found in other viruses (Pfeffer et al. 2004). The cellular functions of most animal miRNAs are unknown.

More than ten years after the discovery of the first miRNA gene, *lin-4* (Chalfie et al. 1981; Lee et al. 1993), we know that miRNA genes constitute about 1%–2% of the known genes in eukaryotes. Investigation of miRNA expression combined with genetic and molecular studies in *Caenorhabditis elegans, Drosophila melanogaster,* and Arabidopsis thaliana have identified the biological functions of several miRNAs (recent review, Bartel 2004). In *C. elegans, lin-4* and *let-7* were first discovered as key regulators of developmental timing in early larval developmental transitions (Ambros 2000; Abrahante et al. 2003; Lin et al. 2003; Vella et al. 2004). More recently *lsy-6* was shown to determine the left–right asymmetry of chemoreceptor expression (Johnston and Hobert 2003). In *D. melanogaster, miR-14* has a role in apoptosis and fat metabolism (Xu et al. 2003) and the *bantam* miRNA targets the gene *hid* involved in apoptosis and growth control (Brennecke et al. 2003). In mouse, *miR-181a* modulates hematopoietic differentiation (Chen et al. 2004), and *miR-196* directs the cleavage of *Hox-B8* transcripts (Yekta et al. 2004).

miRNAs have specificity. In a range of organisms, miRNAs are differentially expressed in developmental stages, cell types, and tissues (Lee and Ambros 2001; Lagos-Quintana et al. 2002; Sempere et al. 2004). In particular, differential expression has been observed in mammalian organs (Lagos-Quintana et al. 2002; Krichevsky et al. 2003; Sempere et al. 2004) and embryonic stem cells (Houbaviy et al. 2003). Estimates in worm show that there are approximately 1,000 molecules of miRNA per cell, with some cells exceeding 50,000 molecules (Lim et al. 2003b). This dynamic range of regulation of miRNA expression underscores the regulatory functional importance of miRNAs.

### The Mechanism of miRNA Action

How do miRNAs pair with their target messages? miRNAs cause the translational repression or cleavage of target messages (Doench and Sharp 2004). Some miRNAs may behave like small interfering RNAs (siRNAs) that direct mRNA cleavage between the nucleotide positions 10 and 11 in the siRNA:mRNA target duplex (Tuschl et al. 1999; Zamore et al. 2000; Elbashir et al. 2001; Hutvágner and Zamore 2002a; Llave et al. 2002b; Martinez et al. 2002; Bartel 2004; Yekta et al. 2004). It appears that the extent of base pairing between the small RNA and the mRNA determines the balance between cleavage and degradation (Hutvágner and Zamore 2002a). Recent examples of cleavage of target messages are, in mouse, *mir-196* guiding cleavage of *Hox-B8* transcripts (Yekta et al. 2004) and, in Epstein Barr virus, *miR-BART2,* a virus-encoded miRNA, guiding the cleavage of transcripts for virus DNA polymerase (gene *BALF5*) (Pfeffer et al. 2004). While cleavage of mRNA is a straightforward process, the details of the mechanism of translational repression are unknown.

The following rules for matches between miRNA and target messages have been deduced from a range of experiments. (1) Asymmetry: experimentally verified miRNA target sites indicate that the 5′ end of the miRNA tends to have more bases complementary to the target than its 3′ end. Loopouts in either the mRNA or the miRNA between positions 9 and 14 of the miRNA have been observed or deduced (Brennecke et al. 2003; Johnston and Hobert 2003; Lin et al. 2003; Vella et al. 2004). Recent experiments show some correlation between the level of translational repression and the free energy of binding of the first eight nucleotides in the 5′ region of the miRNA (Doench and Sharp 2004). However, confirmed miRNA:mRNA target pairs can have mismatches in this region (Moss et al. 1997; Johnston and Hobert 2003). (2) G:U wobbles: wobble base pairs are less common in the 5′ end of a miRNA:mRNA duplex, and recent work shows a disproportionate penalty of G:U pairing relative to standard thermodynamic considerations (Doench and Sharp 2004). (3) Cooperativity of binding: many miRNAs can bind to one gene (Reinhart et al. 2000; Ambros 2003; Vella et al. 2004), and the target sites may overlap to some degree (Doench and Sharp 2004).

Given the overlap between the siRNA and miRNA pathways, it is reasonable to assume that rules of regulation in the siRNA pathway will partly apply to miRNA target recognition (Hutvágner and Zamore 2002b; Boutet et al. 2003; Doench et al. 2003). Lately, detailed characteristics associated with siRNA functionality were identified: low G/C content, a bias towards low internal stability at the3′ terminus, lack of inverted repeats, and strand base preferences (positions 3, 10, 13, and 19) (Jackson et al. 2003; Reynolds et al. 2004). These observations may provide clues for better quantitative description of miRNA:mRNA interaction. Regions adjacent or near to the target site can be important for miRNA specificity. In *lin-41,* a 27-nucleotide (nt) intervening sequence between two consecutive *let-7* sites is necessary for its regulation (Vella et al. 2004). Because of lack of conservation of this 27-nt intervening sequence in *C. briggsae,* incorporation of a corresponding rule is premature.

### Maturation of miRNAs and Assembly in RNA-Induced Silencing Complex

miRNAs are transcribed as longer precursors, termed pre-miRNAs (Lee et al. 2002), sometimes in clusters and frequently in introns (25% of human miRNAs; Table S1). Upon transcription, miRNAs undergo nuclear cleavage by the RNase III endonuclease Drosha, producing the 60–70-nt stem-loop precursor miRNA (pre-miRNA) with a 5′ phosphate and a 2-nt 3′ overhang (Lee et al. 2003). The pre-miRNA is subsequently transported across the nuclear membrane, dependent on the protein exportin 5 (Lund et al. 2003; Yi et al. 2003). Dicer cleaves the pre-miRNA in the cytoplasm about two helical turns away from the ends of the pre-miRNA stem loop, producing double-stranded RNA. A helicase unwinds the cleaved double-stranded RNA in a strand-specific direction (Khvorova et al. 2003; Schwarz et al. 2003).

One of the unwound strands is subsequently incorporated into a ribonuclear particle (RNP) complex, RNA-induced silencing complex (RISC) (Hutvágner and Zamore 2002a; Martinez et al. 2002). Every RISC contains a member of the Argonaute protein family, which tightly binds the RNA in the complex (Hammond et al. 2001; Hutvágner and Zamore 2002a; Martinez et al. 2002; Mourelatos et al. 2002). There are at least eight members of the Argonaute family in mammals (Sasaki et al. 2003), and only a small subset has been functionally characterized. The Argonautes and Dicer bind single-stranded RNA via their PAZ domains (Lingel et al. 2003; Sasaki et al. 2003; Song et al. 2003; Yan et al. 2003), and the known structures of the PAZ domains may have implications for prediction of miRNA targets (Lingel et al. 2003; Song et al. 2003; Yan et al. 2003).

### Association of mRNAs and miRNAs with Fragile X Mental Retardation Protein

Among the prime candidates for miRNA control are the genes that are posttranscriptionally regulated. The mRNA-binding protein fragile X mental retardation protein (FMRP) is involved in the regulation of local protein synthesis (Antar and Bassell 2003) and binds 4% of mRNAs expressed in the rat brain, as tested in vitro (Brown et al. 2001). The loss of function of FMRP causes fragile X syndrome, the most prevalent form of mental retardation (one in every 2,000 children). Over the past three years a number of different groups have identified in vivo mRNA cargoes of FMRP. The Warren and Darnell laboratories have identified ligands by co-immunoprecipitation followed by microarray analysis, complemented by extraction of polyribosomal fractions (Brown et al. 2001). They discovered that FMRP and one of its three RNA-binding domains specifically binds to G-rich quartet motifs (Brown et al. 2001; Darnell et al. 2001; Denman 2003; Miyashiro et al. 2003). Three more studies found that mRNAs containing U-rich motifs bind recombinant FMRP in vitro and associate with FMRP-containing mRNPs in vivo (Chen et al. 2003; Denman 2003). Lastly, antibody-positioned RNA amplification as a primary screen followed by traditional methods identified over 80 FMRP-regulated mRNAs, with a combination of G-quartet and U-rich motifs in their mRNA sequences (Miyashiro et al. 2003).

Independently, FMRP has been shown to be associated with RISC components and miRNAs (Jin et al. 2004). The *Drosophila* homolog of FMRP (FXR) and the Vasa intronic gene were identified as components of RISC (Caudy et al. 2002). More recent studies have proved that mammalian FMRP interacts with miRNAs and with the components of the miRNA pathways including Dicer and the mammalian orthologs of Argonaute (AGO) 1 (Ishizuka et al. 2002; Jin et al. 2004). Given the association of FMRP with Argonaute-containing complexes, we propose and investigate the hypothesis that the cargoes carried by FMRP are also miRNA targets, and we derive hypotheses of specific pairing interactions.

Here, we predict miRNA targets in five vertebrate genomes as a way of facilitating experiments and exploring a number of open questions. What proportion of all genes is regulated by miRNAs? How many genes are regulated by each miRNA? Are specific cellular processes targeted by specific miRNAs or by miRNAs in general? What is the extent of cooperativity in miRNA:mRNA binding?

## Results

### Prediction of miRNA Targets

**Figure 1:**
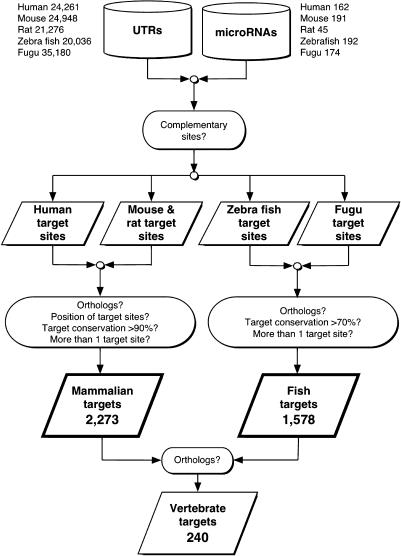
Target Prediction Pipeline for miRNA Targets in Vertebrates The mammalian (human, mouse, and rat) and fish (zebra and fugu) 3′ UTRs were first scanned for miRNA target sites using position-specific rules of sequence complementarity. Next, aligned UTRs of orthologous genes were used to check for conservation of miRNA–target relationships (“target conservation”) between mammalian genomes and, separately, between fish genomes. The main results (bottom) are the conserved mammalian and conserved fish targets, for each miRNA, as well as a smaller set of super-conserved vertebrate targets.

Using currently known mammalian miRNA sequences, we scanned 3′ untranslated regions (UTRs) from the human *(Homo sapiens),* mouse *(Mus musculus),* and rat *(Rattus norvegicus)* genomes for potential target sites. The scanning algorithm was based on sequence complementarity between the mature miRNA and the target site, binding energy of the miRNA–target duplex, and evolutionary conservation of the target site sequence and target position in aligned UTRs of homologous genes. We identified as conserved across mammals a total of 2,273 target genes with more than one target site at 90% conservation of target site sequence (Tables S2 and S3) and 660 target genes at 100% conservation. We also scanned the zebrafish *(Danio rerio)* and fugu *(Fugu rubripes)* fish genomes for potential targets using known and predicted miRNAs (Figure 1; Tables S4 and S5) and identified 1,578 target genes with two or more conserved miRNA sites between the two fish species.

In addition to the analysis of 3′ UTRs, we also scanned all protein-coding regions for high-scoring miRNA target sites. For convenience, these results are reported both as hits in cDNAs (coding plus noncoding; Table S6) and as hits in coding regions (Table S7), with cross-references in the UTR target tables (number of hits in the coding region for each UTR in Tables S2 and S3).

The algorithm and cutoff parameters were chosen to provide a flexible mechanism for position-specific constraints and to capture what is currently known about experimentally verified miRNA target sites: (1) nonuniform distribution of the number of sequence-complementary target sites for different miRNAs; (2) 5′–3′ asymmetry (the complementary pairing of about ten nucleotides at the 5′ end is more important than that of the ten nucleotides at the 3′ end [Doench and Sharp 2004], and the matches near the 3′ end can to a limited extent compensate for weaker 5′ binding); and (3) influence of G:U wobbles on binding (Doench and Sharp 2004). In choosing these parameters, we drew on experience from careful analysis of target predictions in *Drosophila* (Enright et al. 2003) as well as proposed human targets of virus-encoded miRNAs (Pfeffer et al. 2004).

To facilitate evaluation of predicted targets and design of new experiments, we provide methods and results in a convenient and transparent form. We make the miRanda software freely available under an open-source license, so that researchers can adjust the algorithm, numerical parameters, and position-specific rules. We also provide web resources, including a viewer for browsing potential target sites, conserved with or without positional constraints, on aligned UTRs, with periodic updates (http://www.microrna.org, as well as links to these targets from the miRNA registry site RFAM (http://www.sanger.ac.uk; Griffiths-Jones 2004). We provide both high-scoring targets, as strong candidates for validation experiments, and lower-scoring targets, which may have a role in broader background regulation of protein dose. Expression information (see Table S3) for miRNAs and mRNAs provides an additional filter for validation experiments, in addition ranking target sites by complementarity and evolutionary conservation.

### Validation of Target Predictions

Only a small number of target sites of target genes regulated by miRNAs have been experimentally verified, so we sought direct and indirect evidence to help validate or invalidate the proposed set of mammalian targets. (1) We compared predicted targets with experimentally verified targets in mammals, *C. elegans,* and D. melanogaster, as well as their mammalian homologs. (2) We compared predicted target numbers from real and shuffled miRNA sequences and estimated the rate of false-positive predictions. (3) We assessed the enrichment of miRNA targets in mRNAs that are known cargoes of FMRP, an RNA-binding protein known to be involved in translational regulation.

#### Agreement with known targets

We previously used known miRNA sites for the *let-*7 and *lin-4* miRNAs in *Drosophila* to develop the target prediction method and check for consistency (Enright et al. 2003). More recent experimental target identification provides independent control data. Recent work in C. elegans (Vella et al. 2004) has narrowed the originally reported list of six target sites for *let-7* in the UTR of *lin-41* down to three elements, two target sites, and a 27-nt intervening sequence (a possible binding site for another factor). The surviving two target sites have high alignment scores, *S* = 115 and *S* = 110, while the other four sites are below threshold (Enright et al. 2003), fully consistent with the experimental results. As one of the confirmed sites has a single-residue bulge, target prediction methods that require a perfect run of base pairs near the 5′ end of the miRNA would not detect it, while our method does. *lsy-6,* a recently experimentally identified miRNA in *C. elegans,* controls left–right neuronal asymmetry via *cog-1,* an Nkx-type homeobox gene; the *cog-1* gene has a target site in its 3′ UTR, which also has a high score (*S* = 125) and passes the conservation filter.

Experiments in D. melanogaster have identified six new miRNA–target gene pairs: *miR-7* targets the notch signaling genes *HLHm3, HLHm4,* and *hairy,* and *miR-2b* targets the genes *reaper*, *grim,* and *sickle* (Stark et al. 2003). Consistent with these experiments, our target predictions in D. melanogaster (Enright et al. 2003) ranked *HLHm3, hairy,* and *HLHm4* at positions 1, 3, and 7, respectively, in the list of 143 target genes for *miR-7* (Enright et al. 2003). Similarly, our predictions ranked *reaper, grim,* and *sickle* at positions 3, 11, and 19, respectively, among the other 120 predicted target genes for *miR-2c*. We also predicted *miR-6* to target this group of pro-apoptotic genes, with sites that have lower scores than the *miR-2* family but are conserved in *D. pseudoobscura.* Unfortunately, one cannot in general use these predicted and then validated target sites (Stark et al. 2003) for the derivation of new prediction rules, as the set of targets tested is limited to the type predicted and is not exhaustive.

Indirect validation comes from the prediction that mammalian orthologs of some of the known miRNA targets in C. elegans and D. melanogaster are miRNA targets. An example is the proposed conservation of the miRNA–target relationship *lin-4:lin-28* (we use the notation miRNA:mRNA for a miRNA–target pair), first discovered in worm (Moss and Tang 2003): we detect target sites in human *lin-28* for the *lin-4* miRNA homolog *miR-125*. We also confirm the human analog of a *let-7:lin-28* relation predicted in C. elegans (Reinhart et al. 2000). In summary, the predicted target sites on human *lin-28* are *miR-125* (1 site), *let-7b* (2 sites; Moss and Tang 2003), *miR-98* (2 sites), and *miR-351* (1 site). Another known *lin-4* and *let-7* target in C. elegans is *lin-41*. The human homolog of *lin-41* (sequence provided by F. J. Slack, personal communication) and another closely related gene (encoding Tripartite motif protein 2) are predicted as high-ranking targets of *let-7* and *miR-125* (the human homolog of *lin-4*) (see Tables S2 and S3). Another known instance of miRNA target regulation in worms is the regulation of *cog-1* by the *lsy-6* miRNA (Johnston and Hobert 2003). Although there is no obvious homolog of *lsy-6* in mammals, the vertebrate homolog of the target gene *cog-1, nkx-6.1,* is a conserved target for five different miRNAs in our predictions (see Table S2).

The comparison of our results with known targets shows that our method can detect most (but not all) known target sites and target genes at reasonably high rank. However, given the small number of experimentally verified miRNA–target pairs, additional validation tests are desirable, such as statistical tests using randomization of miRNA sequences to estimate false positives.

#### Estimate of false positives

As a computational control of the validity of the prediction method, one can perform a statistical test that attempts to estimate the probability that a predicted site is incorrect. Here, a “false positive” is a predicted target site of a real miRNA on a real mRNA that has passed all relevant thresholds but is incorrect in that it is not biologically meaningful. The statement “not biologically meaningful” is rarely clearly defined, but can reasonably be taken to mean that no functionally effective miRNA:mRNA interaction occurs under conditions of co-expression at physiological concentration, where “functionally effective” is defined in terms of detectable changes of phenotypic attributes.

**Figure 2:**
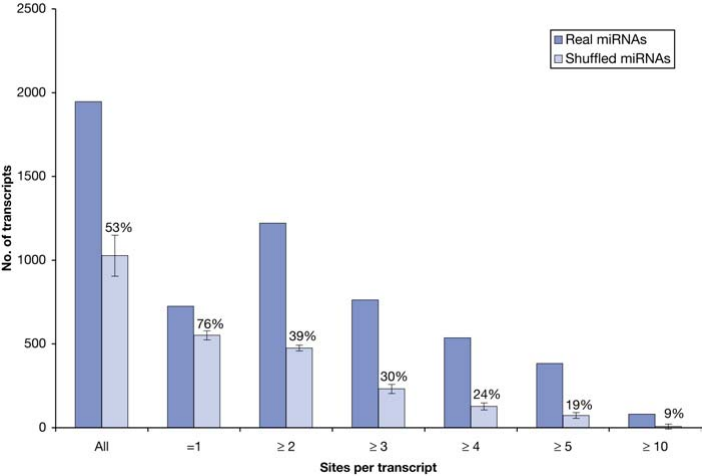
Distribution of Transcripts with Cooperativity of Target Sites and Estimated Number of False Positives Each bar reflects the number of human transcripts with a given number of target sites on their UTR. Estimated rate of false positives (e.g., 39% for ≥2 targets) is given by the number of target sites predicted using shuffled miRNAs processed in a way identical to real miRNAs, including the use of interspecies conservation filter.

Technically, an estimate of the false-positive rate can be obtained by computing (directly or via randomization) the background distribution of scores for biologically non-meaningful miRNA target sites and then deriving the probability that a non-meaningful target site passes all score thresholds, i.e., for a single aggregate score, that the incorrect site has a score *T* > *T_c_,* where *T_c_* is a fixed threshold that may be, in general, different for each miRNA. We chose to estimate the background distribution using shuffled miRNAs obtained by swapping randomly selected pairs of bases of each given miRNA 1,000 times, keeping the nucleotide composition constant. The shuffled miRNA sequences were scanned against human, mouse, and rat 3′ UTR sequences exactly as for the prediction procedure for real miRNA sequences. In the procedure, a miRNA:mRNA match site is predicted to be a target site if it passes three thresholds, *S* > *S_c_* for match score, |Δ*G*| > |Δ*G_c_*| for free energy of duplex formation, and *C* > *C_c_* for conservation, where *C* reflects a binary evaluation of orthology of mRNAs, similarity of position of the site on the mRNA, and a threshold percentage of conserved residues in the two mRNA target sites. Finally, the predicted target sites for a set of shuffled miRNAs are counted and then averaged over a total of ten randomized runs. The percentage of false positives for target transcripts with more than two, three, and four sites is 39%, 30%, and 24%, respectively, using a non-permissive conservation threshold of 100% for target site sequences (Figure 2). In addition, the false-positive rate for single sites with a score of more than 110 is approximately 35%.

To provide a realistic estimate of false positives using randomization, the distribution of scores from random trials (“random-false”) should be similar to the distribution of incorrect (non-meaningful) hits from real trials (“real-false”). The difference between these two distributions is difficult to compute in principle, as very few validated correct predictions are known at present. For human sequences, without any conservation filter, we obtained a total of 2,538,431 predicted target sites for real miRNAs, and, for shuffled miRNAs, on average, 2,033,701 (± 82,172) target sites—a difference of 20%. This difference may be indicative of a biological signal in the raw score *(S)* and energy *(*Δ*G)* calculated by the miRanda algorithm or may be due to different polynucleotide compositions of shuffled miRNAs compared to real miRNAs. Even if this difference represents a real effect, by far the most predictive criterion for accurate target detection is conservation of target sites across species, and not alignment scores or energies (20% compared to a factor of three, see Figure 2; Table S8). As a consequence, the current set of predicted targets rests heavily on the criterion of conservation of miRNA:mRNA match between different species. We believe this to be essentially true for all currently published target prediction methods.

#### Indirect experimental support: FMRP-associated mRNAs

An excellent opportunity to test our target predictions comes from experiments showing the association of mRNAs and miRNAs with proteins involved in translational control, even if these experiments do not provide information on specific miRNA:mRNA pairings. In particular, FMRP, which may regulate translation in neurons, not only associates with hundreds of mRNAs (Brown et al. 2001; Chen et al. 2003; Denman 2003; Miyashiro et al. 2003; Waggoner and Liebhaber 2003) and with miRNAs (Jin et al. 2004), but also associates with components of the miRNA processing machinery, *Dicer,* and the mammalian homologs of AGO1 and AGO2 (Jin et al. 2004). If all FMRP-bound mRNAs are regulated by miRNAs, one should see a large enrichment of predicted targets among such mRNAs. We tested this hypothesis with 397 FMRP-associated mRNAs taken from a number of recent experiments (Brown et al. 2001; Chen et al. 2003; Denman 2003; Miyashiro et al. 2003; Waggoner and Liebhaber 2003).

Are FMRP-bound messages enriched in predicted targets? Using five different datasets (Table S9), we predicted that 74% of FMRP-associated messages are miRNA target genes (294 of 397 mRNAs). This corresponds to an enrichment factor of about five compared to the 59 targets one would expect from our analysis in a randomly chosen set of 397 mRNAs, where 59/397 equals 4,462/29,785 (4,462 predicted mammalian target mRNAs pass the 90% conservation filter for one or more sites per transcript out of a total of 29,785 transcripts). This suggests that in the 397 FMRP target genes, 59 should pass the filters. The enrichment factor does not vary much with the cutoff parameters used in target prediction (data not shown), but is subject to some uncertainty because of potential false-positive predictions. The enrichment of miRNA:FMRP interaction is consistent with the hypothesis that translational control involving FMRP protein is executed in a complex that involves one or more miRNAs interacting with transcripts at specific sites. Note that this analysis supports the validity of target gene prediction, not the identity of the controlling miRNA or the accuracy of specific sites.

An additional validation test involved FMRP cargoes that had been identified in more than one study, using independent experimental methods. For example, the mRNAs of 14 genes (Brown et al. 2001) were overrepresented in both the polyribosome fraction of mouse fragile X cells and in co-immunoprecipitation with mouse brain mRNPs that contain FMRP. Almost all of the 14 genes are predicted targets with more than one conserved site (11 of 12 annotated UTRs; Table S9). In some cases, expression data provide additional support: *postsynaptic density protein 95 (PSD95)–associated (SAPAP4),* a neuron-specific protein, is regulated by many miRNAs highly expressed in rat brain primary cortical neurons (Kim et al. 2004).

In summary, the three validation approaches (retrospective, statistical, and indirect experimental) suggest that the current version of the miRanda algorithm, in spite of clear limitations, can predict true miRNA targets at reasonable accuracy, provided that (1) the targets are detected as conserved and (2) the gene contains more than one miRNA target site or a single high-scoring site (*S* > 110, approximately, including sites with almost perfect complementarity suggestive of mRNA cleavage).

### Overview of Mammalian miRNA Target Genes

#### More than 2,000 mammalian targets.

**Figure 3:**
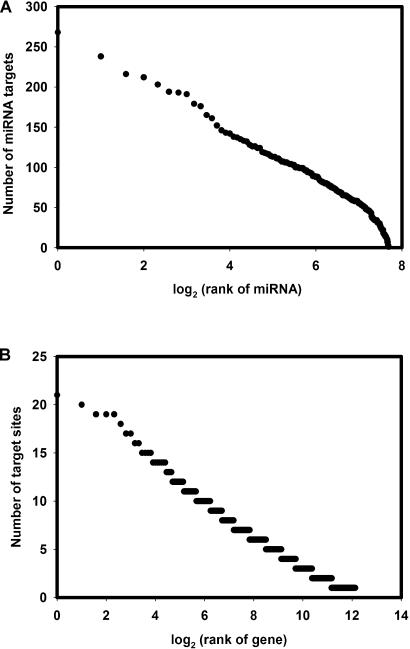
Multiplicity and Cooperativity in miRNA–Target Interactions One miRNA can target more than one gene (multiplicity) (A), and one gene can be controlled by more than one miRNA (cooperativity) (B). The distributions are based on ordered (ranked) lists and decay approximately exponentially (approximate straight line in log-linear plot). (A) Some miRNAs appear to be very promiscuous (top left), with hundreds of predicted targets, but most miRNAs control only a few genes (bottom right). (B) Some target genes appear to be subject to highly cooperative control (top left), but most genes do not have more than four targets sites (bottom right). Although specific values are likely to change with refinement of target prediction rules, the overall character of the distribution may well be a biologically relevant feature reflecting system properties of regulation by miRNAs.

We predicted 2,273 genes as targets with two or more miRNA target sites in their 3′ UTRs conserved in mammals at 90% target site conservation (see Tables S2 and S3). This means we predicted approximately 9% of protein-coding genes to be under miRNA regulation. In addition, we predicted another 2,128 genes with only one target site, but the false-positive rate for these is significantly higher (Figure 2). Of these, the top-scoring 480 genes (*S* > 110) have an estimated false-positive rate comparable to that of genes with multiple sites and thus also are good candidates for experimental verification. Some of the genes with single sites may contain additional sites that we cannot detect for a number of reasons, including truncated UTRs. A significant subset of the total number of single-site target genes (7%) has near complementary single sites. These near complementary sites may indicate cleavage, for which additional sites may not be necessary. The targets listed in Table 1 were selected for variety of function, variation in number of sites, and varied extent of conservation (some are also conserved in fish). Somewhat surprisingly, the number of predicted targets per miRNA varies greatly, from zero (for seven miRNAs) to 268 (for *let-7b*), but the distribution is nonuniform (mean = 7.1, standard deviation = 4.7; Figure 3). This indicates a range of specificity for most miRNAs and suggests that regulation of one message by one miRNA is rare.

**Table 1:**
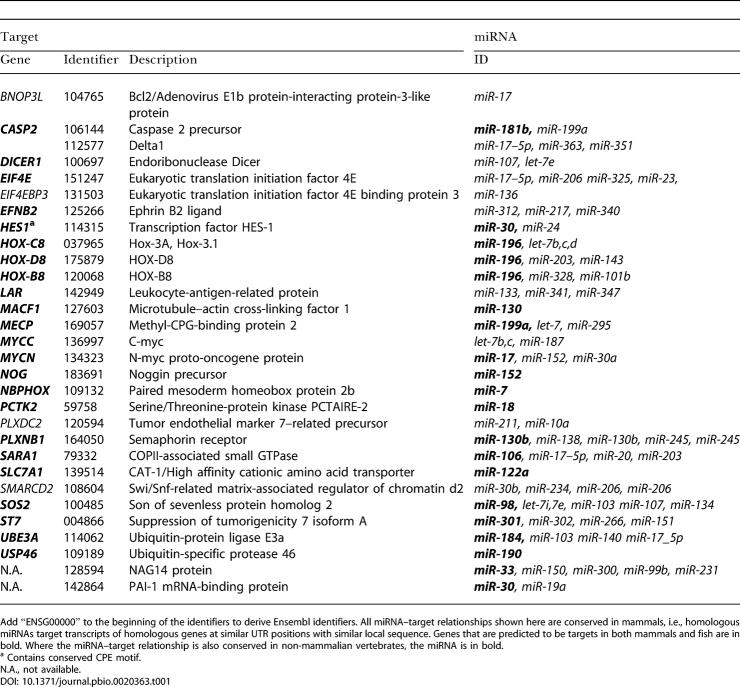
Selection of Predicted miRNA Targets

Add “ENSG00000” to the beginning of the identifiers to derive Ensembl identifiers. All miRNA–target relationships shown here are conserved in mammals, i.e., homologous miRNAs target transcripts of homologous genes at similar UTR positions with similar local sequence. Genes that are predicted to be targets in both mammals and fish are in bold. Where the miRNA–target relationship is also conserved in non-mammalian vertebrates, the miRNA is in bold

^a^ Contains conserved CPE motif

N.A., not available

#### Functional analysis

We analyzed the distribution of functional annotation for all targets of all miRNAs using Gene Ontology (GO) terms (see Materials and Methods; Table S10) and domain annotations from InterPro (Mulder et al. 2003). The target genes reflected a broad range of biological functions (Figure S1). The most enriched GO term was “ubiquitin-protein ligase activity,” with 3.3-fold enrichment (Table S10). Since ubiquitination is a process controlling the quantity of specific proteins in a cell at specific times, miRNA regulation of components of the ubiquitin pathway could increase protein levels. Other overrepresented functional terms were “neurogenesis” (3.2-fold), “protein serine/threonine kinase” (2.5-fold), and “protein-tyrosine kinase activity” (2.5-fold).

The four domains most overrepresented in predicted targets relative to all genes were *Homeobox* domain, 5.3-fold; *KH* domain, 4.0-fold; and *Guanine-nucleotide dissociation stimulator CDC25* domain, 3.4-fold (Figure S1; Table S10). Interestingly, *KH* domains are RNA-binding domains found in a wide range of proteins such as hnRNPk, FMR1, and NOVA-1. In addition to the *Homeobox* domain, other DNA-binding domains and domains associated with chromatin regulation were also enriched, suggesting that miRNAs in animals target the transcription machinery disproportionately, as they do in plants. Another overrepresented domain was *semaphorins* (3.0-fold). The *semaphorins* and *plexins* (semaphorin receptors) are involved in axon guidance, angiogenesis, cell migration, the immune system, and the adult nervous system (Pasterkamp and Verhaagen 2001). Many semaphorins and their receptors are predicted targets of brain-expressed miRNAs (e.g., *let-7c, miR-125b, miR-153, miR-103, miR-323, miR-326,* and *miR-337*). The plexins dimerize with Neuropilin (NP1) to signal the Semaphorin ligand attachment; *neuropilin* is a predicted high-ranking target of *let-7g* and *miR-130,* both brain-expressed miRNAs. A significant proportion of ephrin receptors (seven out of ten genes) and ephrin ligands (five out of seven genes) are predicted targets. The family of *ephrins* is involved in boundary formation, cell migration, axon guidance, synapse formation, and angiogenesis, and the ephrin ligand, *EphA2,* which contains a conserved cytoplasmic polyadenylation element (CPE) motif, is considered to be under translational regulation in axon growth cones (Steward and Schuman 2003). Although many members of the *ephrin* family are predicted targets of brain-expressed miRNAs, they appear to be targeted by different miRNAs, consistent with differential regulation. In *Drosophila,* both *ephrin* and *EphR,* closest to the mammalian B class of the *ephrin* family, also are predicted miRNA targets.

Do specific miRNAs target particular functional groups? We analyzed each miRNA individually for GO term and domain enrichment (Table S10). The targets of some miRNAs were strongly enriched in certain categories, e.g., *miR-105* in “small GTPase mediated signal transduction” (5-fold), *miR-208* in “transcription factor” (6-fold), and *miR-7,* which lies in the intron of the hnRNPk (an RNA-binding protein) gene, in “RNA binding proteins.” Neuronal differentiation of embryonic carcinoma cells by retinoic acid in both mice and humans is coupled to induction of *let-7b, miR-30, miR-98, miR-103,* and *miR-135* (Sempere et al. 2004), and their targets are enriched in “neurogenesis” (3.5-fold). *miR-124a* and *miR-125,* both highly and specifically expressed in brain, preferentially target RNA-binding proteins. Thirty-one new miRNAs *(miR*-*322*–*miR*-*352)* cloned from rat neuronal polyribosomes have a large number of neuronal target genes and share many targets, e.g., *miR-352* and *miR-327* target 5HT-2c, and *miR-340, -328, -326, -331,* and *-333* potentially target beta-catenin, which is implicated in various stages of neural differentiation.

Two highly expressed miRNAs in the thymus, *miR-181a* and *miR-142–3p* are key components of a molecular circuitry that modulates hematopoietic lineage (Chen et al. 2004). Ectopic expression of *miR-181a* causes a 2-fold increase in the cells of the B cell lymphoid lineage. Some of our high-ranking targets for *miR-181a* may provide clues for the mechanism of this effect. Germ cell nuclear factor *GCNF (NR6A1)* (the second-highest-ranked target for *miR-181a*) is expressed in the thymus and bone marrow. *miR-181a* itself is encoded on the antisense strand of an intron of *GCNF*. We also predict that the gene *Bcl11b,* known to affect B cell growth, is a target of *miR-181a,* ranking third, as well as Lim/homeobox protein LHX9, recently found expressed in developing thymus (Woodside et al. 2004).

#### FMRP cargo mRNAs regulated by miRNAs

FMRP is composed of several RNA-binding domains (two *KH* and one RRG) that bind messages. The specific binding motifs for FMRP on messages are incompletely known, but are thought to include G-quartet patterns and/or U-rich sequences (Dolzhanskaya et al. 2003; Ramos et al. 2003). We predicted 294 mRNAs known to be FMRP cargoes as miRNA targets (see Table S9). The most reliable of these (Table 2) reflect high confidence in experimental identification of FMRP association or conservation of target site between mammals and fish.

**Table 2:**
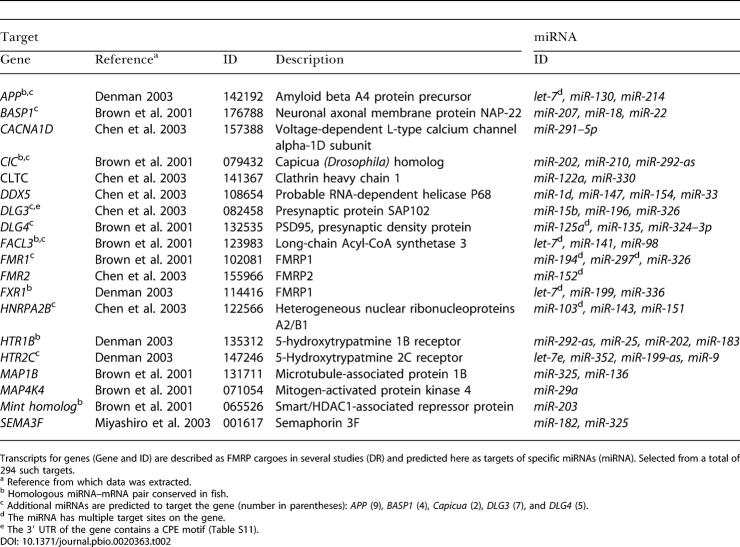
Selected FMRP Cargoes Predicted as miRNA Targets

Transcripts for genes (Gene and ID) are described as FMRP cargoes in several studies (DR) and predicted here as targets of specific miRNAs (miRNA). Selected from a total of 294 such targets

^a^ Reference from which data was extracted

^b^ Homologous miRNA–mRNA pair conserved in fish

^c^ Additional miRNAs are predicted to target the gene (number in parentheses): *APP* (9), *BASP1* (4), *Capicua* (2), *DLG3* (7), and *DLG4* (5)

^d^ The miRNA has multiple target sites on the gene

^e^ The 3′ UTR of the gene contains a CPE motif (Table S11)

#### Alzheimer's disease amyloid protein

**Figure 4:**
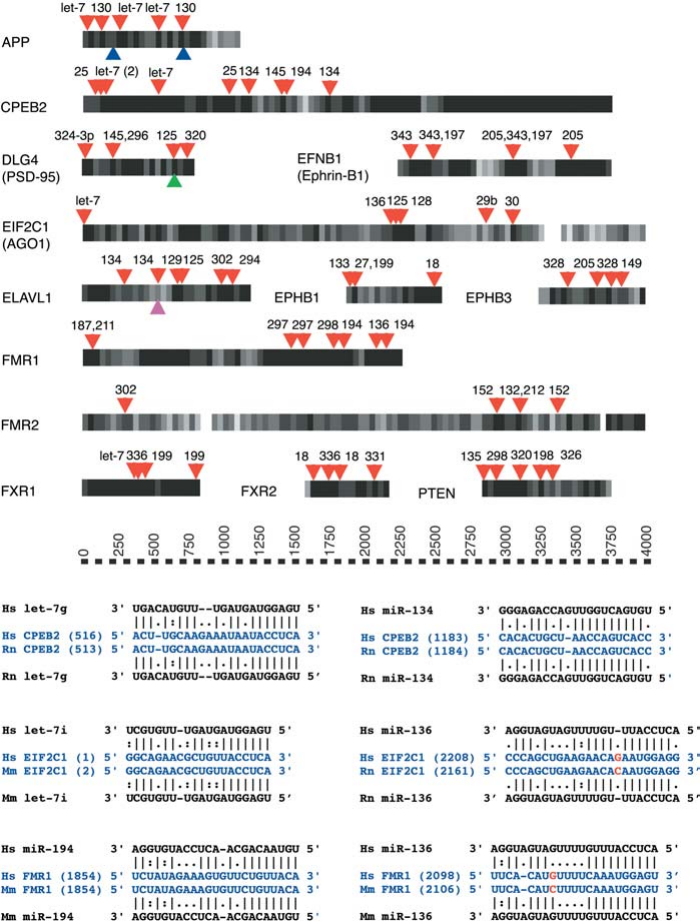
Potential miRNA Target Sites in the 3′ UTRs of Selected Genes Nucleotide sequence conservation between the 3′ UTRs of human and the closest mouse or rat orthologous genes is averaged for each block of 40 base pairs (long rectangles; white indicates 0% identical nucleotides, black indicates 100% identical nucleotides, and grey indicates intermediate values). The positions of target sites for specific miRNAs (triangles above rectangles, with numbers indicating *miR* miRNAs, e.g. “130” is “mir-130”) are, in general, distributed nonuniformly. Sequence motifs other than target sites (triangles below rectangles) are mRNA stability elements (APP), a G-quartet (DLG4), and an AU-rich element (ELAVL1), representing possible protein-binding sites. Detailed alignments between the miRNA and the predicted target sites (arbitrary selection) illustrate, in general, stronger match density at the 5′ end of miRNAs than at the 3′ end, as required by the algorithm and as observed in experimentally validated targets. The nonconserved nucleotides in the target sites are highlighted in red. Gene names map to the following Ensembl identifiers (142192 is ENSG00000142192, etc.): APP, 142192; CPEB2, 137449; DLG4, 132535; EFNB1, 090776; EIF2c1, 092847; ELAVL1, 066044; EPHB1, 154928; EPHB3, 182580; FMR1, 102081; FMR2, 155966; FXR1, 114416; FXR2, 129245; and PTEN, 171862.

Amyloid precursor protein (APP) is an FMRP-bound protein that is translationally regulated. The *APP* transcript contains a 29-nt motif at position 200 in the 3′ UTR that is known to aid destabilization of the *APP* mRNA in certain nutrient conditions and that binds nucleolin, a protein associated with RNPs containing FMRP (Rajagopalan and Malter 2000). In addition, there is an 81-nt sequence at position 630 in the *APP* 3′ UTR that is required for the TGFbeta-induced stabilization of the *APP* mRNA (Amara et al. 1999). We predicted *APP* as a target, with a total score of *S* = 708 with a minimum of eight miRNA sites, including two *let-7* top-ranking sites that are conserved in human, mouse, and rat. One of the predicted miRNA target sites in the *APP* UTR lies in the 81-nt region (Figure 4), and another is within 30 nt of the motif at position 200.

Other APP-interacting proteins, APP-binding family B member 1 *(mir-9, miR-340,* and *miR-135b),* APP-binding family member 2 *(let-7* and *miR-218),* and APP-binding family 2 *(miR-188* and *miR-206)* were also predicted targets, some of which had near exact target site matches. In summary, the *APP* gene appears to be subject to translational regulation by the combinatorial control of a number of different miRNAs.

#### PSD95 and synaptic processes

PSD95 and similar scaffolding molecules, link the NMDA receptor with intracellular enzymes that mediate signaling; this process is involved in the development and maintenance of synaptic function and synaptic plasticity, and interference in this process is implicated in schizophrenia and bipolar disorder (Beneyto and Meador-Woodruff 2003). FMRP binds PSD95 and is required for mGluR-dependent translation of PSD95 (Todd et al. 2003). PSD95 is a high-ranking target of *miR-125, miR-135, miR-320,* and *miR-327,* all of which are either exclusively expressed in brain or enriched in brain tissue (Lagos-Quintana et al. 2002; Krichevsky et al. 2003; Sempere et al. 2004). In particular, large transcript numbers of *miR-125b* are found copurified with polyribosomes in rat neurons in (Kim et al. 2004). *PSD95* has one reported G-quartet in its 3′ UTR at position 648 (Todd et al. 2003), further suggesting it as an in vivo FMRP target. We predicted an additional G-quartet site at position 205–235 in the 3′ UTR of *PSD95*. One of the miRNA *(miR-125)* target sites overlaps with the G-quartets, raising the possibility that miRNAs directly compete with FMRP to bind the message in this location. Likewise, NAP-22, which has three miRNA target sites (see Table S9), has a *miR-207* target site that overlaps with a G-quartet (Darnell et al. 2001).

Other *PSD95* family members are also involved in synaptic processes, in particular, in the integration of NMDA signaling in the synaptic membrane. All *PSD95* family members in mammals (also known as *discs large 1–5*), *SAP90,* and *CamKII* are predicted miRNA targets (see Table S9), as well as *mGluR,* the protein product of which is an agonist that induces the rapid translation of PSD95 (Todd et al. 2003) and three NMDA receptor subunits (see Table S9). These results suggest that miRNAs may be involved in NMDA and glutamate receptor signaling to coordinate and integrate information, with specificity achieved through the combinatorial action of different miRNAs.

### Components of RNPs Regulated by miRNAs

#### FMRP-associated proteins

FMRP binds its own mRNA, implying negative feedback if the binding inhibits FMRP production (Ceman et al. 1999)*.* The fact that miRNAs target transcripts for FMRP and FMRP-binding proteins suggests another negative feedback loop in which high levels of these proteins inhibit their own production (depending, of course, on the concentration of miRNAs and mRNAs) (Figure 4). The genes for six FMRP-associated (not associated at the same time) proteins, hnRNP A1, Pur-alpha, Pur-beta, Staufen, AGO-2, and PABP, are predicted miRNA targets. This indicates that FMRP-containing RNPs are under miRNA regulation. *FXR2,* a gene similar to *FMR1* is also a miRNA target in human, mouse, rat, and fish. Details of the implied feedback regulation and differential control of RNP action remain to be determined.

#### RISC.

Our data suggest that the RNAi–miRNA machinery itself is under miRNA regulation; for example *Dicer* appears to be controlled by *let-7* and *miR-15b; Ago-1* by *let-7* and *miR-29b/c; Ago-2* by *miR-138; Ago-3* by *miR-138, miR-25,* and *miR-103;* and *Ago-4* by *miR-27a/b. Dicer* and two of the Argonautes also are predicted to be targets in both zebrafish and fugu. The *let-7* sites on the 3′ UTR of *Dicer* and *Ago-1* (Figure 4) will accommodate most of the *let-7* variants with similar scores. The variants of *let-7* are expressed in a wide range of tissues and developmental stages, suggesting broad regulation of *Dicer* and *Ago-1* by miRNAs. In contrast, the only miRNA that targets *Ago-2* is *miR-138,* which has so far been cloned only once in the cerebellum (Lagos-Quintana et al. 2002). The target site for *miR-138* has only one mismatch at position 8; this may induce a siRNA-like cleavage of the message (Hutvágner and Zamore 2002a; Doench et al. 2003). *Ago-3* is also a top target for *miR-138,* with only two mismatches in its site. We suggest that some miRNAs targeting this machinery (e.g., *let-7, miR-27, miR-29,* and *miR-103)* are expressed fairly widely, while others (e.g., *miR-138* and *miR-25*) have lower and more restricted expression.

#### Other RNPs.

The highly conserved RNA-binding proteins, ELAV-like proteins (HuR, HuB, HuC, and HuD), contain three RNA-recognition motifs, which bind AU-rich elements in 3′ UTRs of a subset of target mRNAs (Good 1995). These AU-rich elements increase the proteins' cytoplasmic stability and increase translatability (Perrone-Bizzozero and Bolognani 2002). Experiments have identified 18 mRNAs bound to HuB in retinoic-acid-induced cells; of the 14 we were able to map unambiguously, 12 are predicted miRNA target genes: *Elavl1* (known to regulate its own mRNA), *Gap-43, c-fos, PN-1, Krox-24, CD51, CF2R, CTCF, NF-M, GLUT-1, c-myc,* and *N-cadherin* (Tenenbaum et al. 2000). Three of the ELAV-like genes themselves are also targets of a large number of miRNAs (see Tables S2 and S3; Figure 4). This is yet another example of miRNAs predicted to target the bound messages of RNA-binding proteins and of the regulation of RNA-binding genes by miRNAs.

### Cytoplasmic Polyadenylation Binding Proteins Regulated by miRNAs

We predicted all four human cytoplasmic polyadenylation binding proteins (CPEBs) known in mammals as miRNA targets ranked within the top 170 target genes with 6–20 sites in their UTRs (Figure 4; Table S11). Indeed, *CPEB2* is the highest-ranking gene of all transcripts*.* The orthologs to CPEB1 in fish and fly (known as *orb* in D. melanogaster) are also predicted as targets. CPEB is an RNA-binding protein first shown to activate translationally dormant mRNAs by regulating cytoplasmic polyadenylation in *Xenopus* oocytes (Hake and Richter 1994). It also regulates dendritic synaptic plasticity (Mendez and Richter 2001; Richter 2001) and dendritic mRNA transport (Mendez and Richter 2001; Huang et al. 2003) and facilitates transport of mRNAs in dendrites together with kinesin and dynein in RNPs (Huang et al. 2003). CPEB binds to its target message through the CPE motif (UUUUAU), which must be within a certain distance of the hexanucleotide AAUAAA. CPEB keeps messages in their dormant state until phosphorylated, after which it activates polyadenylation (Mendez et al. 2000), thereby activating translation or degradation (Mendez et al. 2002). In addition, CPEB co-fractionates with the postsynaptic density fraction in mouse synaptosomes, consistent with translation of stored mRNAs in dendrites being part of the mechanism of synaptic plasticity. We have three more lines of evidence suggesting the notion that translational regulation by CPEB is linked to miRNA regulation. First, our target list and the list of genes regulated by CPEB significantly overlap. There are nine genes known to be CPEB-regulated, seven of which are predicted targets: *alpha-CAMIIK, Map 2, Inositol 1, 4–5-Triphosphate Receptor type 1, Ephrin A receptor class A type 2, SCP-1,* and *CPEB3* (Mendez and Richter 2001). Second, CPEB is known to self-regulate in D. melanogaster (Tan et al. 2001). The *CPEB1* homolog in fly, *orb,* and CPEBs in vertebrates are predicted miRNA targets. Third, the gene most correlated in expression to the CPEB homolog in D. melanogaster is a Piwi protein (Sting), a member of the Argonaute family (Pal-Bhadra et al. 2002; Stuart et al. 2003) that is involved in translational regulation and in the RISC.

Among the predicted miRNA targets, 115 genes also contained CPE motifs, which were conserved in at least two mammals in the same positions in the UTRs and are therefore candidates for CPEB regulation (Table S11; see Materials and Methods). Our predictions include *HuB, HuR, Eif-4 gamma, DAZ* associated protein 2, *VAMP-2* (known to be posttranscriptionally regulated), Presynaptic protein *SAP102,* and brain-derived neurotrophic factor precursor. Taken together these data suggest that the CPEB genes, the known CPEB-regulated genes, and the predicted CPEB-regulated genes are strong miRNA target candidates and provide rich ground for experimentation.

### Targets of Cancer-Related miRNAs

Deregulated expression of certain miRNAs has been linked to human proliferative diseases such as B cell chronic lymphocytic leukemia (Calin et al. 2002; Lagos-Quintana et al. 2003) and colorectal neoplasia (Michael et al. 2003). Recent analysis of the genomic location of known miRNA genes suggested that 50% of miRNA genes are in cancer-associated genomic regions or in fragile sites (Calin et al. 2004). The miRNAs *miR-15* and *miR-16* are located within a 30-kb region at Chromosome 13q14, a region deleted in 50% of B cell chronic lymphocytic leukemias, 50% of mantle cell lymphomas, 16%–40% of multiple myelomas, and 60% of prostate cancers (Calin et al. 2002). Furthermore, *miR-15* and *miR-16* are down-regulated, or their loci lost, in 68% of B cell chronic lymphocytic leukemias (Calin et al. 2002). Similarly, *miR-143* and *miR-145* are down-regulated at the adenomatous and cancer stages of colorectal neoplasia (Michael et al. 2003), and *miR-155* is up-regulated in children with Burkitt lymphoma (Metzler et al. 2004).

Our method predicted cancer-specific (by annotation) gene targets of *miR-15a, miR-15b, miR-16, miR-143, miR-145,* and *miR-155*. The target genes and their miRNA regulators are as follows: (1) *CNOT7,* a gene expressed in colorectal cell lines and primary tumors (Flanagan et al. 2003) *(miR-15a);* (2) *LASS2,* a tumor metastasis suppressor (Pan et al. 2001) *(miR-15b);* (3) *ING4,* a homolog of the tumor suppressor p33 *ING1b,* which stimulates cell cycle arrest, repair, and apoptosis (Shiseki et al. 2003) *(miR-143);* (4) *Gab1,* encoding multivalent Grb2-associated docking protein, which is involved in cell proliferation and survival (Yart et al. 2003) *(miR-155);* and (5) *COL3A1,* a gene up-regulated in advanced carcinoma (Tapper et al. 2001) *(miR-145).*

*miR-16* has a tantalizing number of high-ranking targets that are cancer associated and specifically involved in the Sumo pathway There is increasing evidence that Sumo controls pathways important for the surveillance of genome integrity (Muller et al. 2004). The first- and fifth-highest-ranked targets of *miR-16* are Sumo-1 activating and conjugating enzymes, respectively. The top two single-site targets for *miR-16* are an *Activin type II receptor* gene (TGFbeta signaling) and *Hox-A5,* both known to be dysregulated at the level of protein expression in colon cancers (Wang et al. 2001). Both of these sites show near perfect complementary matching between *miR-16* and the target genes (indicating possible cleavage). Both of these target genes are also targets for another cancer related miRNA, *miR-15*.

### Targets Conserved between Mammals and Fish

Roughly 55 miRNAs have identical mature sequences in fugu and mammals, and 80 have very similar sequences in the two species; additional fish miRNA sequences can be predicted with confidence based on sequence similarity. It is therefore reasonable to expect that the targets of these probably functionally homologous miRNAs are orthologous genes in the different species. To follow up on this hypothesis, we assessed conservation of mammalian miRNA–target pairs between the 2,273 mammalian and 1,578 fish miRNA targets (with more than one target site per UTR). The analysis yielded 240 target genes conserved between mammals and fish. The number 240 is probably an underestimate because of several factors, including: (1) unfinished annotation of genomes, particularly rat and fugu; (2) ambiguity in assigning orthologs; and (3) lack of UTR information**.**

The full set of conserved target genes between fish and mammals indicates a wide functional range of conserved targets (Table S12). Many *Hox* genes are conserved as targets, including the *miR-196* targets, *Hox-A4:miR-34a, Hox-C9:let-7b* (near prefect complementary match), and *Hox-B5:miR-27b*. Examples from the notch signaling pathway include *miR-30:hairy enhancer of split 1 (Hes1)* and *miR-152:noggin.*

### Targets Conserved between Vertebrates and Flies

Twenty-eight of the 78 identified miRNAs in flies have apparent mammalian homologs. Based on this remarkable conservation across hundreds of millions of years, it is reasonable to expect that there is some conservation of target sites, target genes, and target pathways between flies and humans. Most strikingly we can identify *hox* genes and axon guidance genes as common targets between vertebrates and flies, e.g., *capicua* and *sex combs reduced* (one of the vertebrate homologs of *Hox-A5*). The *hox* gene cluster in *Drosophila* contains high-ranking predicted targets (Enright et al. 2003) of *miR-10* and *miR-iab-4,* and the *hox* gene cluster in mammals contains high-ranking targets of *miR-196*. These miRNAs are themselves located in the *hox* gene region. We predicted *miR-iab-4–3p* to target *abd-B* in *Drosophila,* a gene related to the ancestral *hox-7* cluster, the ancestral parent of many of the predicted targets of *miR-196*. Axon guidance receptors and ligands conserved as targets include *Lar, ephrins,* and *slits*. Human *slit1* is a top target of *miR-218,* which itself is transcribed from the intron of *slit2,* suggesting down-regulation of *slit1* by transcription of *slit2*. We expect that there are many more conserved targets but we are hindered by the difficulty of mapping orthologous genes between human and fish. Future work will elucidate to what extent there are common pathways regulated by common miRNAs between vertebrates and invertebrates.

### Target Sites in Protein-Coding Sequences

Experiments suggest that miRNA target sites in metazoans are preferentially in UTRs, not in coding regions. If this is true, a correct target site prediction method should predict a larger number of targets in UTRs than in coding regions. Alternatively, target sites in coding regions may so far have escaped experimental verification, especially in plants, in which targets of miRNAs in coding regions are the rule, not the exception.

To investigate this issue we computed the average density of target sites for high-scoring targets (*S* > 130) and before application of conservation filters. The statistical assessment of the influence of conservation filters in coding regions would have raised complicated issues, as nearly two-thirds of nucleotides in coding regions are conserved between mammalian genomes to preserve amino acid sequences. Interestingly, we found, on average, 11 pre-conservation target sites per 1 million nucleotides in coding regions, versus 15 such target sites per 1 million nucleotides in UTRs. This is consistent with a stronger “raw” prediction signal in UTRs and may indicate a lower number of biologically relevant target sites in coding regions in mammals, consistent with early experimental findings.

As a guide to experimentation, we report all sites in coding regions with an alignment score above 110 for miRNAs of length up to 20 nt and an alignment score above 130 for miRNAs longer than 20 nt (scores depend on the length). These cutoff scores approximately correspond to a 75% complementary match between miRNA and target, leaving open the question of how many match pairs are needed to lead to translational inhibition in coding regions, by any mechanism. We identified 942 genes that contained such sites in their coding regions. Strikingly, there was only one site with a perfect match, and this was for the imprinted *miR-127,* known to be antisense to the reciprocally imprinted retrotransposon-like gene on the opposite strand (Seitz et al. 2003). Of the 942 genes, 25% have been otherwise identified as targets based on conserved target sites in their UTRs. However, only five genes have targets sites in their UTRs complementary to the same miRNA that targets the coding region (see Table S3, columns H and I). For example, *miR-211* has a near perfect complementary site in the coding region of a gene of unknown function (Ensembl ID ENSG00000134030, containing an *Eif-4 gamma* domain) and also has two conserved “normal” sites in the UTR. Similarly, *miR-198* has a site in the coding region, as well as conserved sites in the UTR region, of a sodium and chloride GABA transporter (Ensembl ID ENSG00000157103). However, we see no trend for miRNAs that have conserved sites in UTRs to have additional sites in the coding region; rather, stronger target sites for a given miRNA tend to be confined either to the UTR or the coding region and are rarely in both.

### Target Sites with Near Perfect Matches in cDNAs

We scanned all cDNAs for high-scoring matches without using conservation to check for high-scoring targets, which we may have missed through strict conservation rules (see Table S6). Over 40 genes contain sites that have near perfect complementarity to a miRNA (*S* >120), and these target genes may be cleaved rather than translationally repressed as in the case of *miR-196* and *Hox-B8*. For example *miR-298,* an embryonic-stem-cell-specific miRNA (Houbaviy et al. 2003), has a near match with *MCL-1,* and *miR-328* (neuronally expressed) has a near match with *LIMK-1,* which is known to be involved in synapse formation and function. *miR-129,* expressed in mouse cerebellum, has a near perfect complementary match with *Musashi-1,* which is an RNA-binding gene essential for neural development, regulated in the cerebellum, and up-regulated in medulloblastoma (Yokota et al. 2004).

### Comparison of miRNA Target Prediction Methods

Recently, several computational methods for the prediction of miRNA targets have been developed (Enright et al. 2003; Lewis et al. 2003; Rajewsky and Socci 2003; Stark et al. 2003; Kiriakidou et al. 2004; Rehmsmeier et al. 2004). Two of these have been applied to mammalian miRNAs, as described in Lewis et al. (2003) and Kiriakidou et al. (2004) *.* We now compare and contrast these two methods with each other and with the current version of our method, as further developed from miRanda 1.0 and as applied to mammalian and vertebrate genomes (Enright et al. 2003). We compare algorithms and target lists, as an aid to the design of experiments.

The three prediction methods share the goal of identifying mRNAs targeted by miRNAs. All three use sequence complementarity, free energy calculations of duplex formation, and evolutionary arguments in developing a scoring scheme for evaluation of potential targets. Results are reported as lists of target sites and lists of target genes containing such sites. The three methods differ, however, in important technical details, such as the datasets of miRNA and UTR sequences and the algorithm and scoring scheme, as well as the report format. We now summarize these technical differences and compare the lists of resulting target genes for a common subset of miRNAs. The interpretation of such comparisons is hampered by the fact that selection criteria and the use of numerical cutoffs differ conceptually, and genomic coverage is nonuniform.

In the first method, Lewis et al. used 79 miRNAs in human, mouse, and rat, seeking targets in a UTR dataset extracted from the June 2003 version of the Ensembl database. The UTR dataset had 14,300 ortholog triplets conserved between human, mouse, and rat and 17,000 ortholog pairs between human and mouse. All annotated UTRs were extended by 2 kb of 3′ flanking sequence. The algorithm required exact complementarity of a 7-nt miRNA “seed” sequence, defined as positions 2–8 from the 5′ end of the miRNA, to a potential target site on the mRNA, followed by optimization of mRNA–miRNA duplex free energies between an extended window of 35 additional bases of the mRNA and the rest of the miRNA. Target genes were ranked using a composite scoring function, which took into account all sites for a particular miRNA on a given mRNA. Conserved miRNA:mRNA pairs were required to involve orthologs of miRNA and mRNA in human, mouse, and rat, but there was no requirement for conservation of target site sequence (beyond the seed match) or position on the mRNA. Using shuffled miRNA sequences, with the constraint that shuffled controls match real miRNAs in relevant sequence properties, the false-positive rate of predictions was estimated to be 50% for target genes conserved between mouse and human, 31% for target genes conserved in human, mouse, and rat, and 22% for target genes identified in fugu as well as mammals. As a final result, Lewis et al. reported 400 conserved target genes for the 79 miRNAs. Among these targets, 107 genes were reported as conserved in the fish fugu.

In the second method, Kiriakidou et al. used 94 miRNAs in human and mouse, seeking targets in a dataset of 13,000 UTRs conserved in mouse and human (from Ensembl, date not given). The algorithm used a 38-nt sliding mRNA window and calculation of miRNA–mRNA duplex free energies, keeping duplexes with energies below −20 kcal/mol. The duplexes were further filtered using a set of requirements regarding matches and loop lengths in certain positions, as derived and extrapolated from experimental tests involving a predicted target site for *let-7b* miRNA on the UTR of the human homolog of worm *lin-28*. The target site sequence was engineered into a Luciferase reporter, followed by sequence variation of the target site and test of an initial set of 15 predictions in the same reporter assay. Using shuffled miRNA sequences, and applying the same rules and parameters, the false-positive rate of predictions was estimated to be 50% for targets conserved between human and mouse. As a final result, Kiriakidou et al. reported 5,031 human targets, with 222 reported as conserved in the mouse.

In the third method (this work), we used 218 mammalian miRNAs and 29,785 transcripts derived from Ensembl (Table 3) and, as a final result, report 4,467 target genes. What are the main differences between these three prediction methods? Comparison of the total number of predicted target genes is not very informative, as different datasets and cutoffs were used. We attempted to remove one of the technical differences, by explicitly comparing reported targets for the same set of 79 miRNAs used by Lewis et al. (although significant differences remained in the sets of UTR sequences used): the overlap of target genes between Kiriakidou et al. (out of 189) and Lewis et al. (out of 400) was 10.6%; the overlap between Lewis et al. (out of 400) and this work (out of 2,673) was 46%; and the overlap between Kiriakidou et al. (out of 189) and this work (out of 2,673) was 49%. In each case the totals (“out of”) are the number of target genes for the common set of 79 miRNAs and the percentage is relative to the smaller set of two compared. The obvious reason for the larger overlap with our results, 46% and 49% respectively, is the larger number of targets in our predictions, which in turn is primarily the result of choice of cutoff.

**Table 3:**
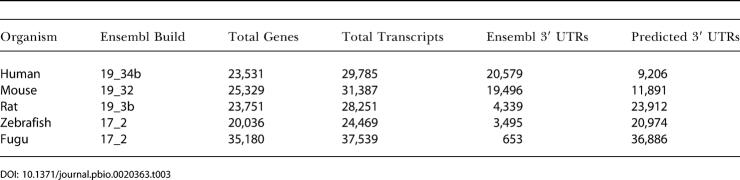
Number of Genes and 3′ UTR Sequences Used for Target Prediction 3′

Direct comparison of the three prediction methods is complicated by the fact that the noticeable differences between the target lists of the three methods are due to the aggregate effects of datasets, algorithm, including selection rules, use of conservation, and cutoffs. The following characteristics of the three methods underlie these differences and should be taken into consideration when choosing targets for experimentation. (1) As to UTR datasets, Lewis et al., with the earliest published report, used a smaller set of UTRs, with some likelihood of false positives as a result of UTR extension. The UTR sets used in this work, the third in terms of publication date, are the most comprehensive and plausibly the most reliable (as of February 2004). (2) As to miRNA datasets, there was an increase from 79 for Lewis et al. to 94 for Kiriakidou et al. to 218 miRNAs used in this work. (3) As to the cooperativity of binding, the scoring system of Lewis et al. evaluated cooperativity of multiple target sites by the same miRNA on a target gene, but disregarded multiple target sites from different miRNAs on one gene; that of Kiriakidou et al. focused on single sites; and that of this work gave high scores to multiple hits on a target gene, no matter whether these hits involved the same miRNA or different miRNAs. These tendencies are not exclusive where scores involve functions of several real numbers, with cutoffs applied to the aggregate score; e.g., our method also allows strong single target sites. (4) As to assessment of false positives using statistical methods based on shuffling, the comparison of percentages is inconclusive, as the statistics of the background distribution of true negatives is not well known. It appears certain, however, from both Lewis et al. and this work, that statistical confidence increases with the extent of conservation among increasingly distant species. (5) As to validation experiments, each of the methods used a different type and set, with mixed overall conclusions. On the reassuring side, there was direct validation of some of the predicted target sites of Lewis et al. and of Kiriakidou et al. using reporter constructs in cell lines. We found some agreement between the sites validated in this way and our predicted targets (details in Table S13), but in some cases we predicted different details of target sites for a given experimentally tested miRNA:mRNA pair. Also, Kiriakidou et al. used a series of such experiments to extrapolate from a set of specific sequence variants to general rules for identification of target sites. However, serious doubts about the validity of any set of rules persist as there is very little in vivo validation in which native levels of specific miRNAs are shown to interact with identified native mRNA targets with observable phenotypic consequences under normal physiological conditions. (6) As to differences in algorithm, one can state opinions about the strengths or weaknesses of each particular algorithm, but the relationship between each prediction method and the actual in vivo process by which miRNAs have functional interactions with their target mRNAs remains unclear or, at best, unproven. In summary, in our view, each of the three methods, including the one in this work, falls substantially short of capturing the full detail of physical, temporal, and spatial requirements of biologically significant miRNA–mRNA interaction. As such, the target lists remain largely unproven, but useful hypotheses.

The predicted targets are useful in practice for the design of experiments as they increase the efficiency of validation experiments by focusing on target lists significantly enhanced in likely targets, compared to random. It is plausible that targets near the top of lists are the most likely to lead to successful experiments. Task-specific filtering of target lists for particular planned experiments is recommended, especially with respect to cooperativity of binding (more than one site for one or more miRNAs on one gene transcript) and coincidence of expression, as new data on expression patterns of miRNAs and mRNAs in different tissues become available. For example, a recommended conservative approach to the design of experiments would use all available expression information and restrict the predicted target genes to those with two or more target sites at normal threshold (*S* > 90) or one target site with a higher threshold (*S* > 110), counting only sites with up to one G:U pair in residues 2–8 counting from the 5′ end of the miRNA.

To take into account the rapid development of this field and the likely close interaction of theory and experiment, we plan to periodically update our prediction method and parameters and make revised target lists available on http://www.microrna.org. Next, we discuss some conceptual consequences of the composition of our target list.

## Discussion

### How Widespread Is the Regulation of Translation by miRNA?

With plausible parameters, we have predicted that close to 9% (2,273 out of 23,531) of all mammalian genes have more than one miRNA target site in their 3′ UTRs, with 1,314 being stronger candidates with more than two target sites. This could well be an underestimate of the total number of genes subject to miRNA regulation, as we have used a conservative conservation filter. On the other hand, not all predicted miRNA–mRNA pairs would have a biological consequence unless both miRNA and mRNA are expressed at the same time in the same cell and at sufficient concentration. The human genome has about 250 miRNA genes, compared to about 35,000 protein genes. Thus, the the determination that about 1% of genes (miRNAs) control the expression of more than 10% of genes is a reasonable first order estimate. It is currently not known if any miRNAs control the expression of miRNA genes, i.e., the progression from miRNA transcript to mature miRNA.

### How Conserved in Evolution Are miRNA Targets?

As many miRNA sequences are detectably conserved across large evolutionary distances, they must be subject to strong functional constraints. These constraints are unlikely to come from single-site interactions with the target, as experimentally validated animal miRNAs rarely have perfectly matched target sites. Plausibly, the evolution of miRNAs is constrained by functional interactions with multiple targets. As a consequence, any compensatory mutation in the miRNA in response to mutations in a target site would be disruptive to the miRNA's interaction with other target sites. Co-evolution of the miRNA sequence and all of its target sequences is therefore a rare event. With these assumptions, the constraints on the local mRNA sequence of individual target sites are weaker than those on the miRNA sequence. We were therefore surprised to observe a substantial number of cases (28.6% of the 2,273 targets) with 100% conservation of target site sequence and with the target sites being within ten nucleotides of each other on the globally aligned UTRs of orthologous genes between mammals.

Lacking more detailed knowledge of miRNA evolution, we draw two operational conclusions. (1) Conservation of target site sequence and position is a practical information filter for predicted target sites, reducing the rate of false positives. (2) It is very likely that new miRNAs have continuously appeared in evolution (Lai 2003) at some non-negligible rate and that the set of targets for any given miRNA has lost or gained members, even between species as close as human and mouse. It is therefore important to develop prediction tools that do not rely on conservation filters or at least allow us to make them weaker. Work on this is in progress.

### Multiplicity and Cooperativity

Regulation by miRNAs is obviously not as simple as one miRNA–one target gene, as perhaps the early examples *(lin-4* and *let-7)* seemed to indicate. The distribution of predicted targets reflects more complicated combinatorics, both in terms of target multiplicity (more than one target per miRNA) and signal integration (more than one miRNA per target gene).

The distribution of the number of target genes (and target sites) per miRNA is highly nonuniform, ranging from zero for seven miRNAs to 268 for *let-7b,* with an average of 7.1 targets per miRNA. It is difficult to describe in detail, beyond the examples discussed in this text and beyond the annotation of target genes in Figure 2 and Table S3, which specific processes appear to be regulated by each miRNA or each set of co-expressed miRNAs. Groups of targets may reflect a reaction, a pathway, or a functional class (see Results). Although all miRNA–target pairs are subject to the condition of synchrony of expression, it is likely that typically one miRNA regulates the translation of a number of target messages and that, in some cases, the target genes as a group are involved in a particular cellular process. This was already known for the case of *lin-4* (Ambros 2003).

The number of miRNA target sites per gene is also nonuniform, with a mean of 2.4. Although we do list target genes with single miRNA sites, there is increasing evidence that, in general, two or more sites are needed in the context of repression of translation. Although the details of these distributions (see Figure 2 and Table S3) depend on technical details, such as uniform cutoff for all miRNAs and evaluation in terms of a particular, imperfect scoring system, the general features of the distributions (see Figure 3) may be generally valid.

We conclude that multiplicity of targets and cooperative signal integration on target genes are key features of the control of translation by miRNAs. Neither multiplicity nor cooperativity is a novel feature in the regulation of gene expression. Indeed, regulation by transcription factors appears to be characterized, at least in eukaryotes, by analogous one-to-many and many-to-one relations between regulating factor and regulated genes (Kadonaga 2004). We are, of course, aware that the control cycles and feedback loops involving miRNAs cannot be adequately described without more detailed knowledge of the control of transcription of miRNA genes, about which little is known at present.

### Mechanisms of miRNA Action

The role of a few animal miRNAs as posttranscriptional regulators of gene expression and, in particular, as inhibitors of translation is well established. However, the molecular mechanism of action is not well understood. Posttranscriptional control of protein levels can be achieved, for example, by cleaving the mRNA, by preventing RNP transport to ribosomes, by stalling or otherwise inhibiting translation on ribosomes, or by facilitating the formation of protein complexes near ribosomes that degrade nascent polypeptide chains. What do our results imply regarding the mechanism of action?

In analogy to plant miRNAs that have near perfect sequence complementarity and facilitate mRNA degradation, our predicted targets with near perfect complementarity between miRNA and mRNA plausibly are involved in mRNA cleavage (e.g., *miR-196* and *miR-138;* see Results). Most of these would involve single target sites. In the case of *Hox-B8,* cleavage has been experimentally shown in mammalian cells (Yekta et al. 2004). We estimate that fewer than 5% of miRNA targets are cleaved as a result of miRNA binding.

Multiple target sites of lesser complementarity are consistent with RNP formation leading to translational inhibition, not mRNA degradation. Although we did predict single miRNA target sites for some genes, most target genes have multiple sites, indicating that cooperative binding (Doench and Sharp 2004) may be essential for formation of inhibitory RNP complexes.

An interesting and somewhat paradoxical feature is seen with mRNAs bound by FMRP, some of which increased and some of which are decreased in polysome fractions in FMRP knock-out mice (Brown et al. 2001). We see no bias in which of these two sets is most enhanced as predicted miRNA targets. This ambiguity not only raises questions about details of FMRP regulation but also raises the possibility that miRNA targets may not always be translationally repressed and may instead be translationally enhanced.

### Improvement of Prediction Rules

Current methods for predicting miRNA targets rely on conservation filters to reduce noise. Although the miRNA–mRNA pairings of experimentally validated targets were carefully used to define prediction rules (Enright et al. 2003; Lewis et al. 2003; Stark et al. 2003), the information content in sequence match scores and free energy estimates of RNA duplex formation appears to be low. What is missing? Perhaps the fine details of experimentally proven target site matches are incorrect, although in some experiments mismatches and insertions have been tested. More plausibly, the rules do not yet capture additional functionally relevant interactions of miRNAs, such as in maturation and transport. Such additional interactions remain to be described in molecular detail, such as interactions with the small RNA processing machinery (Drosha and Dicer) and with the components of RNPs (AGO and FMRP). A first step in this direction is the very recent analysis of the crystal structure of a PAZ domain of a human Argonaute protein, eIF2c1, complexed with a 9-mer RNA oligonucleotide in dimer configuration, which may represent three-dimensional interactions for the 3′ end of a miRNA (and siRNA) complexed, e.g., with Dicer or AGO (Ma et al. 2004). In this structure, each PAZ domain makes close binding contact with nine nucleotides of a single-stranded RNA. The two 3′ terminal nucleotides bind in a pocket through RNA backbone and other contacts. The remaining seven nucleotides bind PAZ through a series of backbone contacts such that nucleotides 3 to 9 are in an RNA helical conformation with bases exposed for base pairing to the second single-stranded RNA. If a 20–21-nt single-stranded RNA is bound to a PAZ domain in the same fashion, the 5′ end would be free for other interactions, such as binding to another protein domain in the RISC or base-pairing to mRNA. The conformational entropy that results when the 3′ end binds to PAZ, because the RNA helix is pre-formed, is consistent with weaker base pairing between miRNA and mRNA at the 3′ end of the miRNA, and stronger base pairing at the 5′ end. The dimeric structure of the PAZ domain (Ma et al. 2004) also raises the tantalizing possibility of cooperative binding of a dimer of two miRNA–PAZ combinations to two target sites on one or more mRNAs. In such an arrangement, seven residues at the 3′ ends of the two miRNAs (residues 3–9, but not the terminal two nucleotides) are paired in antiparallel fashion, with near perfect complementary pairing.

As more details of molecular contacts become available, prediction rules will evolve and improve in accuracy. The following elements are worth considering in the next generation of target prediction rules: (1) details of strand bias as deduced from siRNA experiments (Khvorova et al. 2003), (2) contribution of sequences outside of the mRNA target sites, (3) refinement of position-dependent rules, including different gap penalties for the mRNA and the miRNA, (4) energetics of miRNA–protein binding, starting with PAZ domain interaction, and (5) translation of systematic mutational profiling experiments into scoring rules (Doench and Sharp 2004).

### Principles of Regulation by miRNAs

Although the predicted targets are subject to error (see estimate of false positives) and the prediction rules in need of improvement, several general principles of gene regulation by miRNAs are emerging. (1) Except in cases where a highly complementary match causes cleavage of the target message, miRNAs appear to act cooperatively, requiring two or more target sites per message, for either one or several different miRNAs. (2) Most miRNAs are involved in the translational regulation of several target genes, which in some cases are grouped into functional categories. (3) miRNAs carried in the context of RNPs appear to be sequence-specific adaptors guiding RNPs to particular target sequences. miRNA regulation of cellular messages may therefore range from a switch-like behavior (e.g., cleavage of mRNA message) to a subtle modulation of protein dosage in a cell through low-level translational repression (Bartel and Chen 2004).

These aspects of miRNA regulation complicate the design of experiments aiming at testing target predictions, or, more generally, at discovering biologically meaningful targets. Straightforward experiments that test one target site for one miRNA on one UTR will not be able to disentangle the effects of multiplicity or cooperativity. Tests for multiple sites on one UTR for one miRNA capture aspects of cooperativity (Doench and Sharp 2004), but still do not capture signal integration by diverse miRNAs. The most complicated situation is one in which multiple miRNAs affect multiple genes in combinatorial fashion, with fine-tuning depending on the state of the cell. We look forward to the results of ingenious experiments designed to deal with the complexity of miRNA regulation.

The results of this genome-wide prediction for mammals and fish are meant to be a guide to experiments that will in time elucidate the genetic control network of regulators of transcription, translation/maturation, and degradation of gene products, including miRNAs.

## Materials and Methods

### 

#### miRNA sequences

Mature human and mouse miRNA sequences were obtained from the RFAM miRNA registry (Griffiths-Jones 2004). To cover cases of incomplete data, any mouse miRNA sequence not (yet) described in humans was assumed to be present in human, with the same sequence, and vice versa. Similarly, all mouse miRNAs were assumed to be identical and present in the rat genome. These assumptions are reasonable as sequence identity for known orthologous pairs in human and mouse is, on average, 98% (with 110 out of 146 orthologous sequences being identical). In total, 218 mammalian miRNAs were used. For human target searches, 162 native miRNA sequences were available plus 17 mouse and 39 rat miRNA sequences; for mouse, 191 native, 14 human, and 13 rat sequences; and for rat, 45 native, 159 mouse, and 14 human miRNA sequences.

Mature miRNA sequences for zebrafish and fugu were predicted starting from known human and mouse miRNA precursor sequences (Ambros et al. 2003a). Each precursor sequence was used, in a scan against the zebrafish supercontigs (release 18.2.1) using NCBI BLASTN (version 2.2.6; E-value cutoff, 2.0) (Altschul et al. 1990), to identify a sequence segment containing the potential zebrafish miRNA. The mammalian and fish segments were then realigned using a global alignment protocol (ALIGN in the FASTA package, version 2u65; Pearson and Lipman 1988). After testing the potential fish miRNA precursors for foldback structures (Zuker 2003), the final set of 225 predicted zebrafish miRNAs was selected. The same set of sequences was used for fugu.

#### 3′ UTR sequences

The Ensembl database (Birney et al. 2004) served as the source of genomic data. The Ensembl BioPerl application user interface was used to generate 3′ UTR sequences for all transcripts of all genes from each genome. Some transcripts are alternatively spliced from the same gene, so the total number of genes is smaller than the number of transcripts (Table 3). When no Ensembl annotated 3′ UTR sequences were available, we predicted 3′ UTRs by taking 4,000 bp of genomic sequence downstream of the end of the last exon of a transcript (Table 3). If this predicted region overlapped coding sequence on either strand, we halted 3′ UTR extension at that point.

#### UTR orthology and alignment

Orthology mappings between genes from different genomes were obtained using “orthologue tables” from the EnsMart (Kasprzyk et al. 2004) feature of the Ensembl database. Pairs of orthologous UTRs were aligned with each other using the AVID (Bray et al. 2003) alignment algorithm to facilitate analysis of conservation of position and sequence of target sites. In total, 26,205 human transcripts, representing 15,869 genes, were mapped to both mouse and rat transcripts. For zebrafish, 11,442 transcripts, representing 10,909 genes, were mapped to fugu transcripts and 11,306 transcripts mapped to human transcripts (10,063 genes).

#### miRNA target prediction

The miRanda algorithm (version 1.0; Enright et al. 2003) was used to scan all available miRNA sequences for a given genome against 3′ UTR sequences of that genome derived from the Ensembl database and—tabulated separately—against all cDNA sequences and coding regions. The algorithm uses dynamic programming to search for maximal local complementarity alignments, corresponding to a double-stranded antiparallel duplex. A score of +5 was assigned for G:C and A:T pairs, +2 for G:U wobble pairs, and −3 for mismatch pairs, and the gap-open and gap-elongation parameters were set to −8.0 and −2.0, respectively. To significantly increase the speed of miRanda runs, in calculating the optimal alignment score at positions *i, j* in the alignment scoring matrix, the gap-elongation parameter was used only if the extension to *i, j* of a given stretch of gaps ending at positions *i–1*, *j* or *j–1*, *i* (but not of stretches of gaps ending at *i–k, j* or *j, i–k* for *k* > 1) resulted in a higher score than the addition of a nucleotide–nucleotide match at positions *i, j*. Removal of this restriction with the availability of more computing power would result in a moderate increase in average loop length, but the advantages of this would probably be superceded by overall refinement of target prediction rules. Importantly, complementarity scores at the first eleven positions, counting from the miRNA 5′ end, were multiplied by a scaling factor of 2.0, so as to approximately reflect the experimentally observed 5′–3′ asymmetry; for example, G:C and A:T base pairs contributed +10 to the match score in these positions. The value of the scaling factor at each position is an adjustable parameter subject to optimization as more experimental information becomes available. Because of the ongoing discussion about the rules for target prediction, target genes (a total of 490) that contained target sites with more than one G:U wobble in the 5′ end are flagged in the Table S2. The thresholds for candidate target sites were *S* > 90 and Δ*G* < −17 kcal/mol, where *S* is the sum of single-residue-pair match scores over the alignment trace and Δ*G* is the free energy of duplex formation from a completely dissociated state, calculated using the Vienna package as in Enright et al. (2003).

After finding optimal local matches above these thresholds between a particular miRNA and the set of 3′ UTRs in each genome, we asked whether target site position and sequence for this miRNA were conserved in the 3′ UTRs of orthologous genes, i.e., between human and mouse or rat, or between fugu and zebrafish. The alignments of target sites were generated transitively (UTR→miRNA→UTR) via a shared (or homologous) miRNA. We required that the positions of pairs of target sites in two species fall within ±10 residues in the aligned 3′ UTRs. Conserved target sites with sequence identity of 90% or more (human versus mouse or rat) and 70% or more (zebrafish versus fugu) were selected as candidate miRNA target sites and stored in a MySQL database. Using human as the reference species, we predicted 10,572 conserved target sites (conserved in either mouse or rat) in 4,463 human transcripts, of which 2,307 transcripts of 2,273 genes contained more than one target site. Similarly, using zebrafish as a reference species, we predicted 7,057 conserved target sites (conserved in fugu) in 4,820 zebrafish transcripts.

To focus on the strongest predictions, conserved target sites for each miRNA were sorted according to alignment score, with free energy as the secondary sort criterion. In cases where multiple miRNAs targeted the same site on a transcript (or within 25 nt of a site), only the highest scoring, lowest energy miRNA was reported for that site.

#### Functional analysis of targets.

To facilitate surveys of target function and analysis of functional enrichment, InterPro domain assignments (Mulder et al. 2003) and GO (molecular function hierarchy) mappings (Ashburner and Lewis 2002) for all human genes were obtained using EnsMart. For each functional class derived from either source, we calculated its degree of under- or overrepresentation, *F**_class_**,* using the log-odds ratio of the fraction of annotated target genes with the same class *(F**_1_**)* and the fraction of all annotated Ensembl human genes with that class *(F**_2_**):*



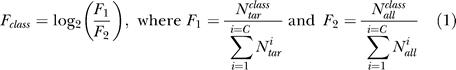



Here, *N* represents the number of genes of a given functional class for either target genes *(N_tar_)* or all genes *(N_all_),* and *C* represents the total number of functional classes. To eliminate bias from small counts we did not report assignments that were present in less than 1% of all annotated target genes (*F_1_* ≤ 0.01 or *F_2_* ≤ 0.01).

#### Randomized trials

For each random experiment all miRNAs were shuffled by randomly swapping two bases of a miRNA 1,000 times. These shuffled sequences were then searched against human, mouse, and rat 3′ UTR sequences in the same way described for the main analysis, including analysis of conservation of target site sequence and position in orthologous 3′ UTRs. A total of ten randomized experiments were performed. Counts were averaged across all experiments, and the standard deviation and other statistical measures were calculated.

#### Analysis of FMRP-associated mRNAs

We compiled a list of 464 gene identifiers of FMRP-associated mRNAs from five different publications (Brown et al. 2001; Chen et al. 2003; Denman 2003; Miyashiro et al. 2003; Waggoner and Liebhaber 2003). Among the 464 gene identifiers, 397 identifiers were mapped to the corresponding genes in our 3′ UTR dataset. The remaining 67 genes were not mapped because their published identifiers were obsolete, primarily because of their Affymetrix probeset identification numbers. To identify miRNA regulation of the 397 FMRP-associated mRNAs, these genes were then compared with the set of predicted miRNA targets.

#### CPE motif prediction.

We predicted CPE motifs in human, mouse, and rat UTRs. We used a search pattern using four criteria: (1) presence of the CPE motif UUUUAU, (2) presence of the hexanucleotide AAUAAA, (3) the CPE and the hexanucleotide motif being within 100 nucleotides of each other, and (4) the conservation of these motifs and the positions of the motifs in the mouse ortholog (Mendez and Richter 2001).

## Supporting Information

Figure S1 Overrepresentation of the GO and Interpro Domains (347 KB PDF).

Table S1 Human miRNAs in Introns (25 KB XLS).

Table S2 Predicted Mammalian miRNA Targets by Gene (8.0 MB XLS).

Table S3 Predicted Mammalian miRNA Targets by miRNA (17.0 MB XLS).

Table S4 Predicted Fish Targets by Gene (5.6 MB XLS).

Table S5 Predicted Fish Targets by miRNA (9.8 MB XLS).

Table S6 High-Scoring miRNA Matches in Human cDNAs (601 KB XLS).

Table S7 High-Scoring miRNA Matches in Human Coding Regions (512 KB XLS).

Table S8 Estimate of False Positives (23 KB XLS).

Table S9 Predicted Targets That Are Associated with FMRP (678 KB XLS).

Table S10 Function of Targets by Interpro and GO Mapping (357 KB XLS).

Table S11 Target Genes That Contain Predicted CPE Motifs (529 KB XLS).

Table S12 Conserved Vertebrate Target Genes (621 KB XLS).

Table S13 Overlap of the Predicted Targets with Validated Gene Targets from Lewis et al. (2003) and Kiriakidou et al. (2004)
(68 KB XLS).

## Abbreviations

AGO: Argonaute
APP: amyloid precursor protein
CPE: cytoplasmic polyadenylation element
CPEB: cytoplasmic polyadenylation binding protein
FMRP: fragile X mental retardation protein
GO: Gene Ontology
miRNA: microRNA
nt: nucleotide
PSD95: postsynaptic density protein 95
RISC: RNA-induced silencing complex
RNP: ribonuclear particle
siRNA: small interfering RNA
UTR: untranslated region
