# Correction: Human MicroRNA Targets

**DOI:** 10.1371/journal.pbio.0030264

**Published:** 2005-07-12

**Authors:** Bino John, Anton J Enright, Alexei Aravin, Thomas Tuschl, Chris Sander, Debora S Marks

In *PLoS Biology,* volume 2, issue 11: DOI: 10.1371/journal.pbio.0020363

We incorrectly named the products of two genes, gamma actin and betaAPP, as FMRP ligands because of a misreading of Table 1 from [1]. BetaAPP is incorrectly referred to as an FMRP ligand in Tables 2 and S9, gamma actin in Table S9 only. This does not change the use of FMRP binding as a way to support the target predictions, as the statistics remain virtually the same; the enrichment of FMRP targets within the predicted microRNA target set changes from 5.31 to 5.26 as a result of our misclassification. In addition, it does not change the fact that betaAPP is a predicted microRNA target. Our Web site at http://www.microrna.org/ contains the updated information with changes flagged. We thank Dr. Denman for pointing out this error to us.
