# Supplementary figures and images for: Human MicroRNA Targets
Source: PLoS Biol. 2004 Oct 5;2(11):e363. doi: 10.1371/journal.pbio.0020363 (PMC521178; doi:10.1371/journal.pbio.0020363)

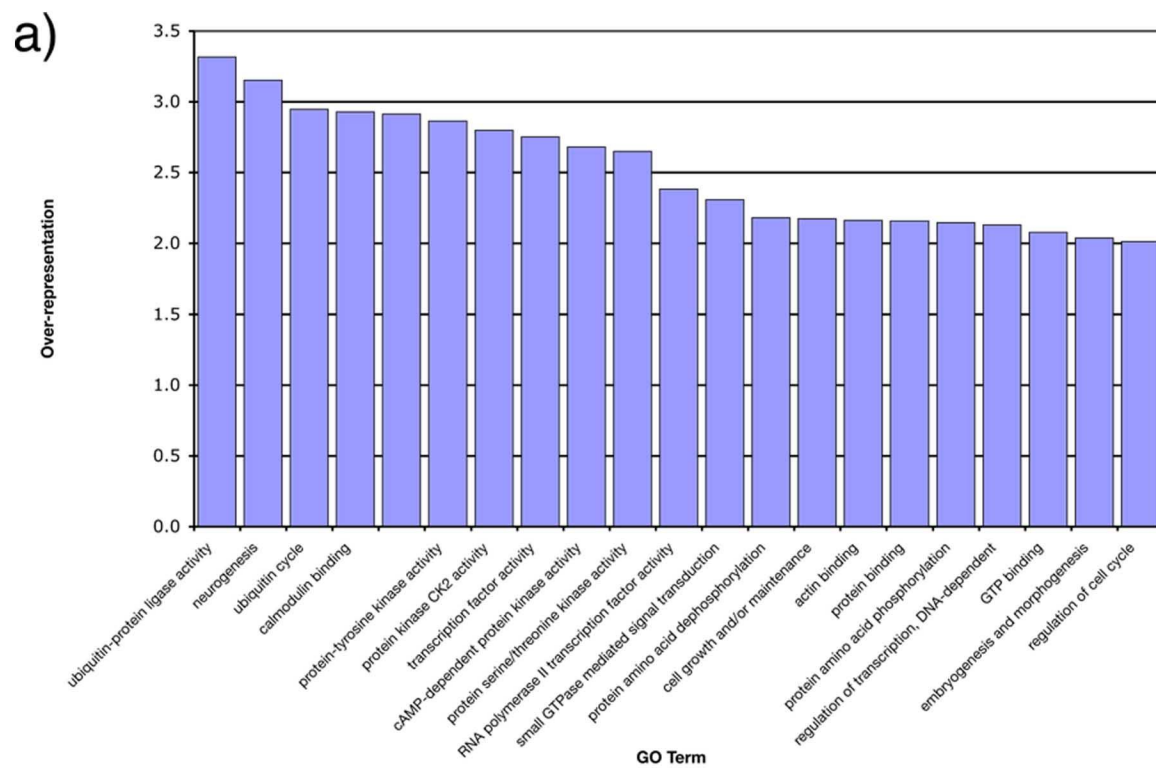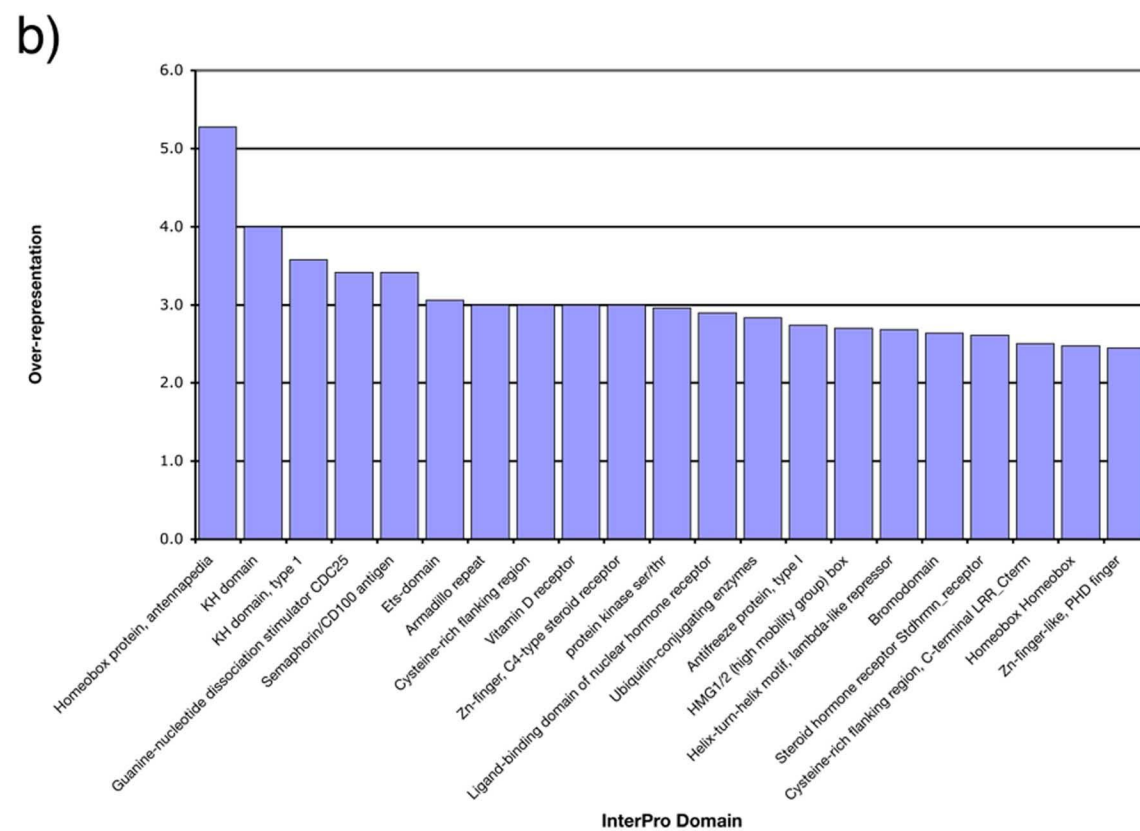

Figure 1 (S13): The over representation of the GO and Interpro domains.

Supplement: Figure S1 — (347 KB PDF). [file pbio.0020363.sg001.pdf]
